# Comparison Between a Single-Lead ECG Garment Device and a Holter Monitor: A Signal Quality Assessment

**DOI:** 10.1007/s10916-024-02077-9

**Published:** 2024-05-27

**Authors:** Luca Neri, Ivan Corazza, Matt T. Oberdier, Jessica Lago, Ilaria Gallelli, Arrigo F.G. Cicero, Igor Diemberger, Alessandro Orro, Amir Beker, Nazareno Paolocci, Henry R. Halperin, Claudio Borghi

**Affiliations:** 1 Department of Medicine, Division of Cardiology, Johns Hopkins University, 1721 East Madison Street Traylor Hall 901, Baltimore, MD 21205 USA; 2 Department of Medical and Surgical Sciences, University of Bologna, Bologna, Italy; 3 Cardiology Unit, IRCCS Azienda Ospedaliero-Universitaria di Bologna, Bologna, Italy; 4 Cardiovascular Medicine Unit, IRCCS Azienda Ospedaliero-Universitaria di Bologna, Bologna, Italy; 5 Institute for Biomedical Technologies, National Research Council (ITB-CNR), Segrate, Italy; 6 Accurate Group S.p.A., L’Aquila, Italy; 7 Department of Biomedical Engineering, Johns Hopkins University, Baltimore, MD USA; 8 Department of Biomedical Sciences, University of Padova, Padova, Italy; 9 Department of Radiology, Johns Hopkins University, Baltimore, MD USA

**Keywords:** Wearable devices, Electrocardiogram, Signal quality, Holter monitor

## Abstract

Wearable electronics are increasingly common and useful as health monitoring devices, many of which feature the ability to record a single-lead electrocardiogram (ECG). However, recording the ECG commonly requires the user to touch the device to complete the lead circuit, which prevents continuous data acquisition. An alternative approach to enable continuous monitoring without user initiation is to embed the leads in a garment. This study assessed ECG data obtained from the YouCare device (a novel sensorized garment) via comparison with a conventional Holter monitor. A cohort of thirty patients (age range: 20–82 years; 16 females and 14 males) were enrolled and monitored for twenty-four hours with both the YouCare device and a Holter monitor. ECG data from both devices were qualitatively assessed by a panel of three expert cardiologists and quantitatively analyzed using specialized software. Patients also responded to a survey about the comfort of the YouCare device as compared to the Holter monitor. The YouCare device was assessed to have 70% of its ECG signals as “Good”, 12% as “Acceptable”, and 18% as “Not Readable”. The R-wave, independently recorded by the YouCare device and Holter monitor, were synchronized within measurement error during 99.4% of cardiac cycles. In addition, patients found the YouCare device more comfortable than the Holter monitor (comfortable 22 vs. 5 and uncomfortable 1 vs. 18, respectively). Therefore, the quality of ECG data collected from the garment-based device was comparable to a Holter monitor when the signal was sufficiently acquired, and the garment was also comfortable.

## Introduction

Wearable electronics have advanced to the point of being comprehensive health monitoring devices [1, 2] and are being combined with other technologies to enhance their capabilities. Current wearable devices are capable of simultaneously recording multiple biosignals such as oxygen saturation, heart rate, and heart rate variability [3–8]. Some models are even able to capture single-lead electrocardiogram (ECG) recordings. However, this function requires that the subject completes the lead circuit by holding the smartwatch case with their opposite hand, thus making the measurement dependent on inconvenient operator action for signal acquisition.

As an alternative, the YouCare device (AccYouRate Group S.p.A., L’Aquila, Italy), a garment with embedded polymer-based electrodes and Bluetooth connectivity, provides the opportunity for continuous single-lead ECG acquisition. Towards establishing the YouCare device as a reliable option for ECG acquisition, its performance was compared to that of a Holter monitor, the clinical standard for wearable ECG monitoring. It was hypothesized that the quality of the signals captured by the YouCare device and Holter monitor are similar.

## Methods

### Overview

Thirty ambulatory patients were subject to 24-hour cardiac rhythm monitoring with the YouCare device and a 3-lead Holter monitor, simultaneously. During the study, the patients performed activities of daily living. All subjects who met the inclusion/exclusion criteria (Table 1) were equipped with the YouCare device, its associated smartphone, and a Holter monitor for 24 h.

**Table 1:** *Eligibility criteria*

*Inclusion criteria*
• Subjects aged ≥ 18 years and ≤ 90 years old, • Subjects with heart rhythm diseases or under screening for the assessment of possible arrhythmias or other heart diseases. • Subjects who have the capability to communicate, to make themselves understood, and to comply with the study’s requirements, • Subjects agree to participate in the study and having dated and signed the informed consent form,
*Exclusion criteria*
• Subjects who have difficulties in wearing the garment such as movements impairments or dermatological reactions to fabric or other materials, • Any medical or surgical condition that would limit the patient’s adherence to the study protocol, • Extreme body habitus, • Subjects who are not able to understand the scope of the study.

The protocol and overall study were approved by an ethics committee (Internal code: 156/2022/Disp/AOUBo by the Comitato Etico Area Vasta Emilia Centro - CE-AVEC – Bologna, Italy), registered on the Italian Ministry of Health website, and on clinicaltrials.gov (Identifier NCT05771142). The study was conducted in accordance with the Declaration of Helsinki, and each participant provided written informed consent.

### YouCare System

**Fig. 1:**
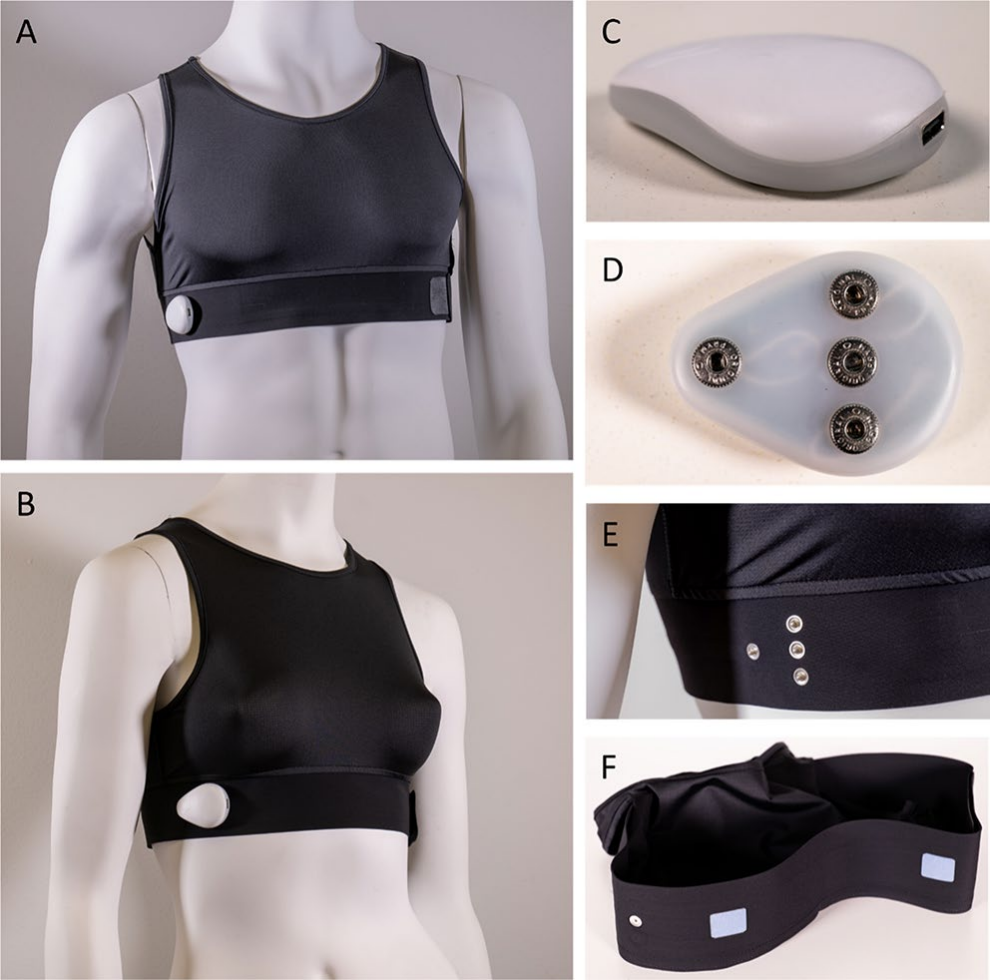
The YouCare device is a garment with polymer-based sensors directly integrated in the garment fabric. When in direct contact with the skin, the device can record a single-lead ECG through sensors in the belt around and below the chest. There are two versions, one for men (**A**) and one for women (**B**) with different sizes. The garment is connected to the control unit, that works as an acquisition and transmission module, (**C**, **D**) through four snaps (**E**). Polymer-based sensors provide contact with the skin (**F**)

The YouCare device (AccYouRate Group S.p.A.; L’Aquila, Italy; Fig. 1) is a crop top garment made of cotton and ceramic with integrated polymer-based electrodes and an acquisition module for data recording and processing. The garment contains 3 polymer-based electrodes that, when in contact with the skin, allow the acquisition of a single-lead ECG. Two of the electrodes are located close to the diaphragm just below the major pectoral muscles (Fig. 1F). A third electrode is positioned on the back of the chest belt, and it has the function of the right leg lead used to reduce the noise and artifacts present on the other two electrodes. The control unit, anchored to the garment via four metal snap fasteners (Fig. 1D-E), has an ECG sampling rate of 246 Hz and collects and sends the data to a smartphone via Bluetooth for storage.

In addition to the one-lead ECG, the garment is paired with a control unit (Fig. 1C) that has an accelerometer, a gyroscope, and body temperature sensor. A respiration waveform is derived from the ECG. YouCare garments are offered in varied sizes and custom fit for women and men (Fig. 1A, B). Garment size is important to ensure continuous sensor contact with the skin, leading to the best signal quality.

### ECG Holter Monitor

The Holter recording system (SEER 1000 GE Healthcare, Chicago, Illinois) features three leads with an ECG sampling rate of 256 Hz, 0.05–70 Hz response, and 12-bit resolution. The system uses standard disposable silver/silver chloride (Ag/AgCl)–gelled electrodes. The electrode-skin connection was reinforced with medical tape to ensure stable contact.

### Assessment and Validation of Signal Quality

ECG signal qualities from both devices were evaluated according to two independent approaches:

1. Qualitative Assessment: ECG signal quality was evaluated by a team of three expert cardiologists and classified according to three categories: “Good” (all major ECG features - P, QRS, and T - are visible for diagnostic purposes), “Acceptable” (the QRS is visible), and “Not Readable” (mostly noise with no ECG waveforms clearly visible).
2. Quantitative Validation: R-R interval comparison between the YouCare device and the Holter monitor were performed after extracting 30 consecutive minutes of data where the quality was at least “Acceptable”. The time distances between corresponding R waves of each device were classified as either within the measurement error (of 8 milliseconds, as determined from error propagation rules [9]), or over the measurement error. The R-R interval comparison was not performed for two patients (#’s 2 and 25) because thirty consecutive minutes of stable signals were not available. Only the longest, uninterrupted recordings were analyzed, and the corresponding segments of the Holter ECG were isolated for comparison. The analysis was performed with Python and its libraries (i.e.: Numpy v.1.17.3, Pandas v1.3.4, Neurokit2 v0.1.7 [10]) in conjunction with ANScovery (SparkBio S.r.l., San Lazzaro di Savena, Bologna, Italy) [11].

### Patient Surveys

A survey of the patients in the study was performed via follow-up phone interview in which patients were asked to rate both the YouCare and Holter devices on a scale of four levels of comfort: very comfortable, comfortable, uncomfortable, very uncomfortable. Four patients were unreachable via the telephone and three did not participate due to a language barrier.

## Results

The thirty patients studied had a mean age of 55 years (range 20–82) and sixteen were women. (Table 2). The average patient was 167 centimeters tall and 70 kg with a body mass index of 25 and a waist circumference of 92 centimeters. The mean heart rate was 75 beats per minute and average blood pressure was 128 over 79 millimeters of mercury. Twelve patients had a history of transient ischemic attack or stroke, twelve patients had a history of palpitations, tachycardia, or extrasystole, six had other cardiac pathologies including mitral insufficiency, and eighteen had a history of chronotropic drug therapy.

**Table 2:** Baseline characteristics of the study population. Values are average (standard deviation)

			Patients (*N* = 30)
Age [years]			55.3 (20.2); Range 20–82
Females [n [%]]			16 [53.3%]
Height [centimeter]			166.7 (7.6)
Weight [kilograms]			69.6 (14.4)
Body Mass Index [kilograms/meter^2^]			24.9 (4.2)
Waist circumference [centimeter]			91.7 (13.7)
Resting Heart Rate [beats per minute]			74.6 (16.1)
Systolic Blood Pressure [millimeters of mercury]			127.5 (13.4)
Diastolic blood pressure [millimeters of mercury]			78.5 (8.7)
Transient Ischemic Event or Stroke (# of patients)			12
Palpitations, Tachycardia, or Extrasystole (# of patients)			12
Other cardiac pathologies (e.g., mitral insufficiency) (# of patients)			6
Chronotropic Drug Therapy (# of patients)			18

### Assessment and Validation of Signal Quality

Connectivity issues between the control unit and smartphone led to data loss. In 17 cases, lost data was less than 1 h, in 5 cases lost data was between 1 and 10 h, and in 8 cases lost data was between 10 and 20 h. Data lost due to Bluetooth disconnections was not considered in the following analyses and is thus also not shown in tables or figures.

**Fig. 2:**
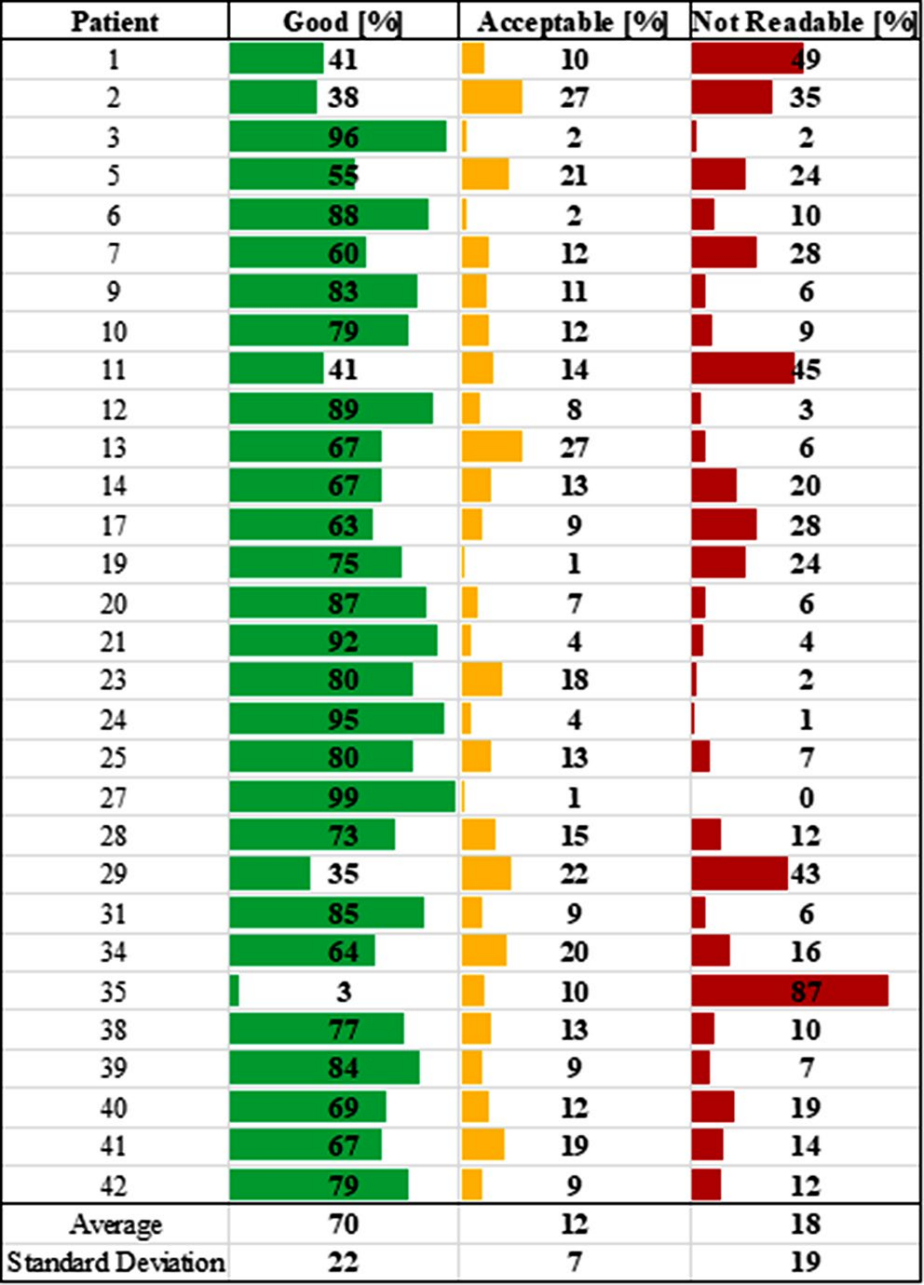
ECG quality assessment using data recorded with the YouCare device for each patient. These percentages do not include data lost due to Bluetooth disconnections

**Fig. 3:**
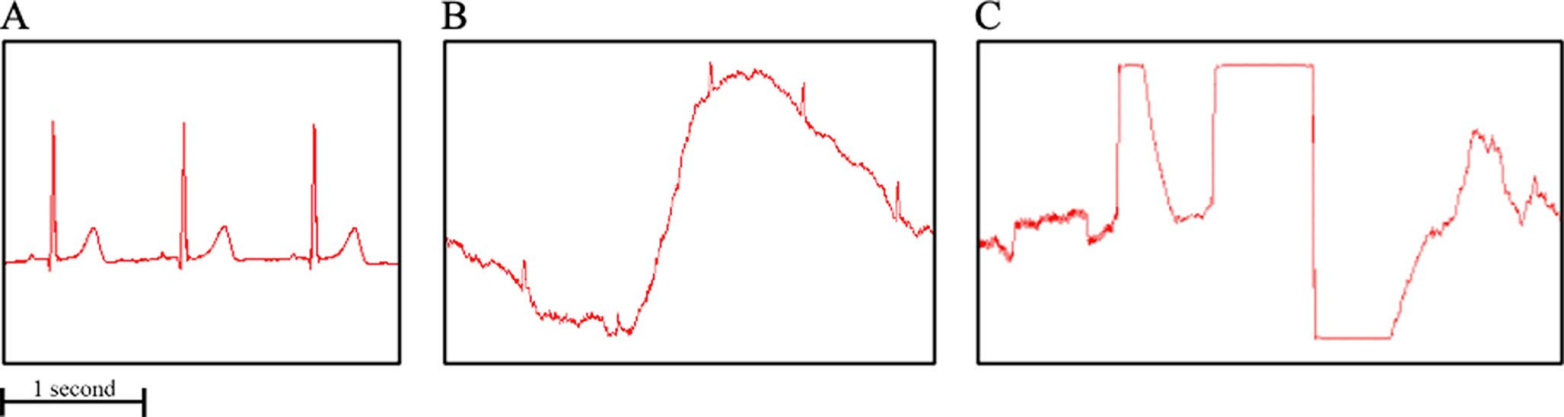
Representative ECG signals recorded with the YouCare classified as: **A**. “Good” – all ECG waveform features (P, QRS, and T) are visible; **B**. “Acceptable” – the QRS is visible; **C**. “Not Readable” – mostly noise with no ECG waveforms clearly visible

From data that was acquired without loss due to Bluetooth disconnections, experts’ assessments determined that signal quality from the YouCare device was “Good” 70% of the time, “Acceptable” 12% of the time, and “Not Readable” 18% of the time (Fig. 2). For four patients, the signal was “Good” at least 90% of the time, and for twenty-four patients, the signal was “Good” at least 60% of the time. Signals from the Holter monitor were “Good” 99% of the time, “Acceptable” 1% of the time, and “Not Readable” 0% of the time. Representative ECG signals for the YouCare device and based on the three categories are shown in Fig. 3.

**Fig. 4:**
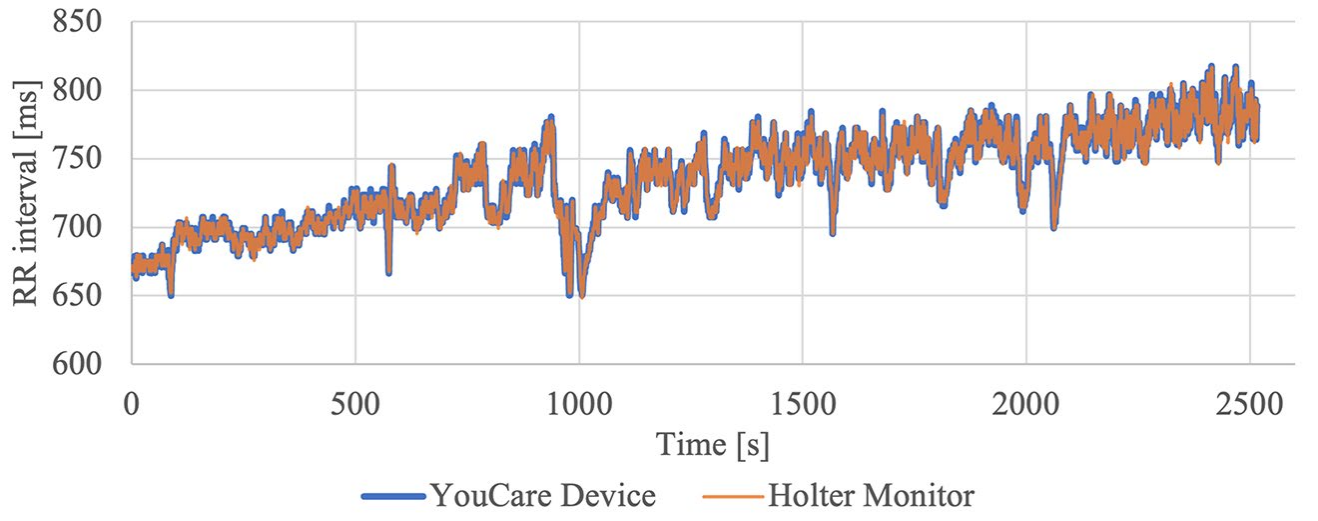
Example of overlapped tachograms from the YouCare device and the Holter monitor

**Fig. 5:**
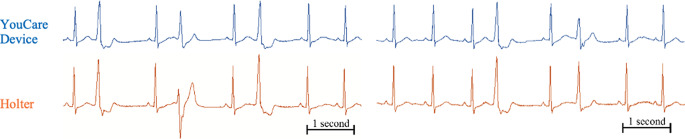
Representative ECG signals with arrhythmias from the YouCare device (above, in blue) and Holter monitor (below, in orange)

There was an R wave overlap between the devices that occurred within the measurement error (≤ 8ms) during 99.4% of cardiac cycles, and outside the measurement error (> 8ms) during 0.6% of cardiac cycles (Table 3). An example of the tachogram is shown in Fig. 4, and representative arrhythmic beats as recorded by each device are shown in Fig. 5.

**Table 3:** Temporal distances between YouCare and Holter devices’ R waves. Patients 2 and 25 were not analyzed because they did not have thirty consecutive minutes of stable signals. ms is milliseconds

	*R* wave sync [%]	
Patient	<= 8 ms	> 8 ms
1	98,7	1,3
3	99,9	0,1
4	99,0	1,0
5	100	0
6	100	0
7	99,9	0,1
8	100	0
9	99,9	0,1
10	98,9	1,1
11	99,6	0,4
12	100	0
13	98,4	1,6
14	99,4	0,6
15	99,6	0,4
16	99,9	0,1
17	98,4	1,6
18	99,9	0,1
19	100	0
20	99,9	0,1
21	99,5	0,5
22	98.7	1.3
23	100	0
24	100	0
26	99,9	0,1
27	100	0
28	99,5	0,5
29	92,8	7,2
30	100	0

### Patient Surveys

**Fig. 6:**
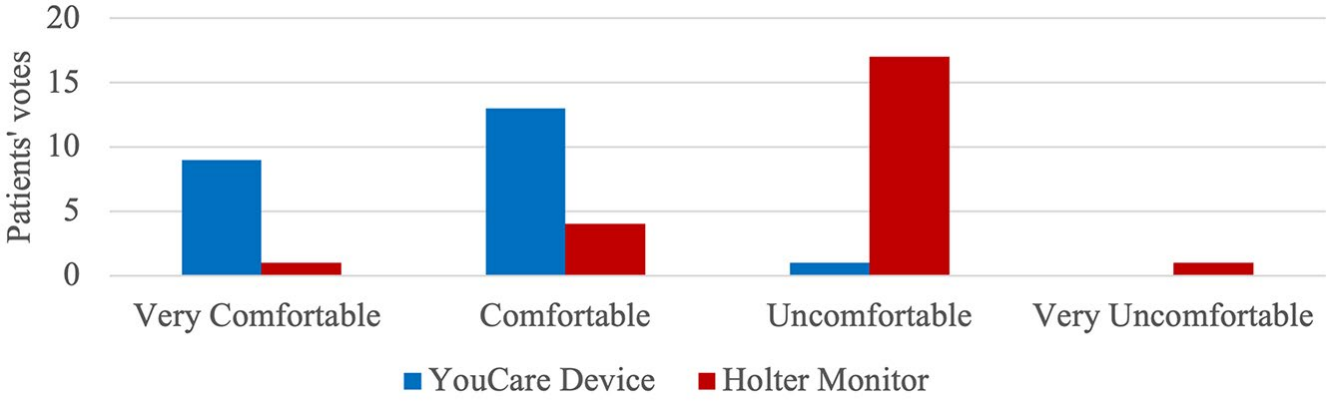
Comfort comparison between the YouCare and Holter devices

The YouCare device was classified as very comfortable by 9 patients, comfortable by 13 patients, uncomfortable by 1 patient, and none considered it very uncomfortable (Fig. 6). On the other hand, the Holter monitor was classified as very comfortable by 1 patient, comfortable by 4 patients, uncomfortable by 17 patients, and 1 considered it very uncomfortable.

## Discussion

This is the first study to evaluate the YouCare device relative to a Holter monitor on patients during their activities of daily living. This is also the first study to detail the comfort of a device for continuous ECG recording, relative to that of a Holter monitor.

Data show the potential of the garment system as a tool for long-term monitoring due to its high comfort and general ability to capture major ECG features. The YouCare device is likely more comfortable due to its integrated sensors that eliminate the need for wires; however, it is important to note that signal acquisition issues related to electrode-skin contact arise, in part, due to a necessary compromise between usability and comfort [12]. In the case of the YouCare device, the increased comfort relative to a Holter monitor came at the expense of intermittent disruptions to electrode-skin contact, accounting for 18% of the signals not being readable, on average.Bluetooth disconnections were also an issue though they accounted for less than an hour of data loss in most patients and were not considered in the analysis because Bluetooth connectivity is an issue apart from ECG signal quality. When the signal was captured without noise, there was an exceptionally reliable overlap of R-waves with most differences being attributed to the use of unique filters and lead configuration inconsistencies between the devices.

Two other studies have evaluated the performance of ECG recording garments relative to Holter monitors. The trial with the OMshirt involved twenty-four hour monitoring of the garment in parallel with the Holter and reported an agreement of around 60% for detecting the QRS complex, and 47% of recordings had some intermittent noise [3]. These findings are consistent with ours in that major ECG features could be detected when noise was minimal, but the continuity of quality signals remains an issue. Another study involving the “hitoe” electrode embedded in a garment showed significant differences between the experimental device and the Holter’s signal-to-noise ratio during the four activities of living that were studied with torso twisting being the least favorable [13]. The study highlights the difficulty in collecting reliable signals during the complex movements that may be experienced during activities of daily living.

This study has several limitations. First, the YouCare and Holter devices were worn simultaneously, potentially resulting in the Holter leads and cables interfering with the electrode-skin contact of the YouCare device. Therefore, it is expected that the YouCare device would have less noise and surface connectivity issues if it were tested in isolation. Second, it was not possible to determine why Bluetooth disconnections occurred or what activity was going on when electrode-skin contacts were not sufficient to maintain a quality signal. For example, poor ECG signals for patients 1, 5, 22, and 29 are potentially due to patient-related factors such as the activities performed by the subjects during the 24 h, possible electrode-garment interactions, and sleep positions that could have reduced the electrode-skin contacts. Such findings are crucial for improving future iterations of the YouCare device. Third, the study was not designed to assess differences between men and women despite obvious differences in upper torso anatomies with specific regards to body hair and breasts. Nonetheless, the YouCare garment was designed with sex-specific considerations, and each patient received a garment that was custom-fitted. Fourth, although individuals with cardiac irregularities (arrhythmias and cerebrovascular and other cardiac issues) were included in the study population, this study did not evaluate device performance in the context of detecting diseases such as arrhythmias. Fifth, this study also did not investigate device performance in the context of other cardiovascular and cardiac devices that may share the same anatomic region and be used in tandem such as left ventricular assist devices or subcutaneous implantable cardioverter defibrillators. A future study is necessary to quantify disease detection, determine potential conflicts with other cardiovascular and cardiac devices, and enable certification of the garment system as a medical device in the United States. Sixth, the R-wave was the only major landmark that was investigated. That is, it has not yet been quantified what capability the YouCare device has in capturing more subtle ECG features such as the P- and T-waves and ST-segment magnitude.

Future development is necessary to improve the continuity of high-quality signal acquisition. Such development may include improved communication between the controller and smartphone or better contacting electrodes. Together, such a device in conjunction with improved ECG analysis techniques [14], including artificial intelligence, have the potential to revolutionize the detection of cardiac arrhythmias and disease in the general population [15] and may even enable the detection of subclinical conditions [5].

## Conclusion

The quality of single-lead ECG data collected from a novel garment-based device was shown to be comparable to a Holter monitor during periods when there was adequate signal acquisition. The garment was also found to be comfortable. Therefore, the garment performs similarly to a Holter monitor and may be a practical means of collecting single-lead ECGs without user actuation to touch the device and thus complete the lead circuit. However, improvements are necessary to ensure signal acquisition is uninterrupted.

## Data Availability

The data presented in this study are available on request from the corresponding author. The data are not publicly available due to the privacy policies of Ethics Committee Area Vasta Emilia Centro - CE-AVEC – Bologna.

## References

[1] Dagher, Lilas, Hanyuan Shi, Yan Zhao, and Nassir F. Marrouche. 2020. Wearables in cardiology: Here to stay. Heart Rhythm 17: 889–895. 10.1016/j.hrthm.2020.02.023. 32354455 10.1016/j.hrthm.2020.02.023

[2] Gargiulo, Gaetano D., and Ganesh R. Naik, ed. 2022. *Wearable/Personal Monitoring Devices Present to Future*. Singapore: Springer Singapore. 10.1007/978-981-16-5324-7.

[3] Steinberg, Christian, François Philippon, Marina Sanchez, Pascal Fortier-Poisson, Gilles O’Hara, Franck Molin, Jean-François Sarrazin, et al. 2019. A Novel Wearable Device for Continuous Ambulatory ECG Recording: Proof of Concept and Assessment of Signal Quality. *Biosensors* 9. Multidisciplinary Digital Publishing Institute: 17. 10.3390/bios9010017. 10.3390/bios9010017 PMC6468449 30669678

[4] Cao, Rui, Iman Azimi, Fatemeh Sarhaddi, Hannakaisa Niela-Vilen, Anna Axelin, Pasi Liljeberg, and Amir M. Rahmani. 2022. Accuracy Assessment of Oura Ring Nocturnal Heart Rate and Heart Rate Variability in Comparison With Electrocardiography in Time and Frequency Domains: Comprehensive Analysis. Journal of Medical Internet Research 24: e27487. 10.2196/27487. 35040799 10.2196/27487 PMC8808342

[5] Duncker, David, Wern Yew Ding, Susan Etheridge, Peter A. Noseworthy, Christian Veltmann, Xiaoxi Yao, T. Jared Bunch, and Dhiraj Gupta. 2021. Smart Wearables for Cardiac Monitoring-Real-World Use beyond Atrial Fibrillation. Sensors (Basel, Switzerland) 21: 2539. 10.3390/s21072539. 33916371 10.3390/s21072539 PMC8038592

[6] Huhn, Sophie, Miriam Axt, Hanns-Christian Gunga, Martina Anna Maggioni, Stephen Munga, David Obor, Ali Sié, et al. 2022. The Impact of Wearable Technologies in Health Research: Scoping Review. JMIR mHealth and uHealth 10: e34384. 10.2196/34384. 35076409 10.2196/34384 PMC8826148

[7] Mannhart, Diego, Mirko Lischer, Sven Knecht, Jeanne du Fay de Lavallaz, Ivo Strebel, Teodor Serban, David Vögeli, et al. 2023. Clinical Validation of 5 Direct-to-Consumer Wearable Smart Devices to Detect Atrial Fibrillation: BASEL Wearable Study. *JACC: Clinical Electrophysiology*. 10.1016/j.jacep.2022.09.011. 10.1016/j.jacep.2022.09.011 36858690

[8] Basza, Mikołaj, Bartosz Krzowski, Paweł Balsam, Marcin Grabowski, Grzegorz Opolski, and Lukasz Kołtowski. 2021. An Apple Watch a day keeps the doctor away? Cardiology Journal 28: 801–803. 10.5603/CJ.2021.0140. 34985118 10.5603/CJ.2021.0140 PMC8747830

[9] Taylor, John R. 2024. An Introduction to Error Analysis: The Study of Uncertainties in Physical Measurements, Second Edition. *University Science Books*. https://uscibooks.aip.org/books/introduction-to-error-analysis-2nd-ed/. Accessed January 11.

[10] Makowski, Dominique, Tam Pham, Zen J. Lau, Jan C. Brammer, François Lespinasse, Hung Pham, Christopher Schölzel, and S. H. Annabel Chen. 2021. NeuroKit2: A Python toolbox for neurophysiological signal processing. *Behavior Research Methods* 53: 1689–1696. 10.3758/s13428-020-01516-y. 10.3758/s13428-020-01516-y 33528817

[11] Corazza, I., G. Barletta, P. Guaraldi, A. Cecere, G. Calandra-Buonaura, E. Altini, R. Zannoli, and P. Cortelli. 2014. A new integrated instrumental approach to autonomic nervous system assessment. Computer Methods and Programs in Biomedicine 117: 267–276. 10.1016/j.cmpb.2014.08.002. 25168777 10.1016/j.cmpb.2014.08.002

[12] Nigusse, Abreha Bayrau, Desalegn Alemu Mengistie, Benny Malengier, Granch Berhe Tseghai, and Lieva Van Langenhove. 2021. Wearable Smart Textiles for Long-Term Electrocardiography Monitoring—A Review. *Sensors* 21. Multidisciplinary Digital Publishing Institute: 4174. 10.3390/s21124174. 10.3390/s21124174 PMC8234162 34204577

[13] Tsukada, Yayoi Tetsuou, Miwa Tokita, Hiroshige Murata, Yasuhiro Hirasawa, Kenji Yodogawa, Yu-ki Iwasaki, Kuniya Asai, et al. 2019. Validation of wearable textile electrodes for ECG monitoring. Heart and Vessels 34: 1203–1211. 10.1007/s00380-019-01347-8. 30680493 10.1007/s00380-019-01347-8 PMC6556171

[14] Neri, Luca, Matt T. Oberdier, Antonio Augello, Masahito Suzuki, Ethan Tumarkin, Sujai Jaipalli, Gian Angelo Geminiani, Henry R. Halperin, and Claudio Borghi. 2023. Algorithm for Mobile Platform-Based Real-Time QRS Detection. *Sensors* 23. Multidisciplinary Digital Publishing Institute: 1625. 10.3390/s23031625. 10.3390/s23031625 PMC9920820 36772665

[15] Neri, Luca, Matt T. Oberdier, Kirsten C. J. van Abeelen, Luca Menghini, Ethan Tumarkin, Hemantkumar Tripathi, Sujai Jaipalli, et al. 2023. Electrocardiogram Monitoring Wearable Devices and Artificial-Intelligence-Enabled Diagnostic Capabilities: A Review. *Sensors* 23. Multidisciplinary Digital Publishing Institute: 4805. 10.3390/s23104805. 10.3390/s23104805 PMC10223364 37430719

